# Appendectomy during the COVID-19 pandemic in Italy: a multicenter ambispective cohort study by the Italian Society of Endoscopic Surgery and new technologies (the CRAC study)

**DOI:** 10.1007/s13304-021-01126-z

**Published:** 2021-07-04

**Authors:** Alberto Sartori, Mauro Podda, Emanuele Botteri, Roberto Passera, Ferdinando Agresta, Alberto Arezzo, the CRAC Study Collaboration Group, M. Guerrieri, M. Ortenzi, F. Cavallo, M. Zese, D. Prando, E. Restini, P. Cianci, P. Millo, R. Brachet Contul, A. Serrao, F. Abatini, D. F. Altomare, A. Picciariello, G. Chetta, F. Lattanzio, V. Tonini, A. Gori, E. Jovine, L. Mastrangelo, L. Sartarelli, A. Frena, A. Malpaga, F. Bertelli, G. Pignata, J. Andreuccetti, S. Sanna, B. Lares, R. Sechi, N. Cillara, A. Pisanu, D. Delogu, G. Ciaccio, M. Farulla, M. Casati, L. Laface, M. De Luca, D. Russello, S. Latteri, M Longoni, E. Masci, S Vigna, F. C. Campanile, N. Foti, P. Lepiane, A. Balla, F. Cantore, V. Raveglia, F. Borghi, G. Giraudo, A. Verzelli, A Budassi, A. Patriti, D. Foghetti, U. Montin, L. Amadio, G. Anania, C. Bombardini, Niccolò Fabbri, Carlo Feo, F. Cianchi, A. Manetti, M. Lucchese, E. Soricelli, G. Ceccarelli, M. Patiti, M. Frascio, C. Stabilini, M. Filauro, A. Barberis, M. Troian, C. Nagliati, R. Campagnacci, A. Maurizi, S. Berti, A. Gennai, A. Marvaso, D. D’Antonio, C. V. Feo, N. Fabbri, L. Mazzola, F. Selvaggi, S. Carini, F. Costanzo, L. Boccia, A. Pascariello, N. Perrotta, M. Celiento, E. Opocher, M. Giovenzana, M. Stella, F. Ferrara, L. Boni, E. Abate, C. Da Lio, V. Valli, R. Gelmini, F. Serra, M. Piccoli, D. Gozzo, A. Gattolin, D. Sasia, A. Balani, B. Petronio, P. G. Calò, G. L. Canu, E. Contarini, G. Piatto, N. Vettoretto, M. Caprioli, M. Braga, M. F. Chiappetta, P. Maida, P. Tammaro, G. De Palma, M. Milone, V. Bottino, A. Canfora, F. Selvaggi, G. Bagaglini, A. Agrusa, M. Barone, A. Mirabella, M. V. Marino, G. Gulotta, G. Romano, M. Sorrentino, S. Ferfoglia, V. Papagni, S. Eramo, C. Boselli, M. Basti, V. Caracino, G. Moretto, M. Inama, P. Capelli, L. Conti, A. Muratore, M. M. Cuoghi, A. Zerbinati, S. Corso, M. C. Vasino, M. Montuori, F. Fidanza, A. Lucchetta, A. Giuliani, G. Dinatale, F. Zanzi, A. Guariniello, S. Bonilauri, G. Frazzetta, M. Garino, C. Marafante, A. Gioffrè, S. R. Del Monte, G. Sganga, P. Fransvea, M. Grande, L. Siragusa, G. Sica, M. Paola, D. G. Passantino, Marco Catani, F. Ricci, E. Lauro, E. Facci, D. Parini, M. F. Armellino, G. Argenio, A. Porcu, T. Perra, P. Bordoni, F. Fleres, A. Parisi, S. Rossi, R. Saracco, D. Bono, T. Viora, F. Orlando, A. Ferrero, A. P. Fontana, P. De Paolis, D. Visconti, F. Quaglino, F. Festa, S. Palagi, G. Lo Secco, M. Morino, M. E. Allaix, A. Salzano, G. Tirone, M. Motter, G. Zanus, N. Passuello, M. Massani, R. Tutino, N. Manzini, S. Terranova, R. Merenda, S. Nordio, S. Zonta, F. Lovisetto, A. Guglielmi, T. Campagnaro, E. Amedeo, M. Scollica, P. Amodio, D. Giannotti, S. Olmi, A. Oldani

**Affiliations:** 1 Department of General Surgery, Ospedale Di Montebelluna, Montebelluna, Italy; 2 Department of Emergency Surgery, Azienda Ospedaliero-Universitaria Di Cagliari, Policlinico Universitario Di Monserrato “Duilio Casula” University of Cagliari, Cagliari, Italy; 3 General Surgery, ASST Spedali Civili Di Brescia, Montichiari, Italy; 4 Division of Nuclear Medicine, University of Torino, Torino, Italy; 5 Department of General Surgery, Ospedale Civile, Adria, Italy; 6 Department of Surgical Sciences, University of Torino, corso AM Dogliotti 14, 10126 Torino, Italy

**Keywords:** COVID-19 Pandemic, Appendicitis, Appendectomy, Machine learning

## Abstract

Major surgical societies advised using non-operative management of appendicitis and suggested against laparoscopy during the COVID-19 pandemic. The hypothesis is that a significant reduction in the number of emergent appendectomies was observed during the pandemic, restricted to complex cases. The study aimed to analyse emergent surgical appendectomies during pandemic on a national basis and compare it to the same period of the previous year. This is a multicentre, retrospective, observational study investigating the outcomes of patients undergoing emergent appendectomy in March–April 2019 vs March–April 2020. The primary outcome was the number of appendectomies performed, classified according to the American Association for the Surgery of Trauma (AAST) score. Secondary outcomes were the type of surgical technique employed (laparoscopic vs open) and the complication rates. One thousand five hundred forty one patients with acute appendicitis underwent surgery during the two study periods. 1337 (86.8%) patients met the inclusion criteria: 546 (40.8%) patients underwent surgery for acute appendicitis in 2020 and 791 (59.2%) in 2019. According to AAST, patients with complicated appendicitis operated in 2019 were 30.3% vs 39.9% in 2020 (*p* = 0.001). We observed an increase in the number of post-operative complications in 2020 (15.9%) compared to 2019 (9.6%) (*p* < 0.001). The following determinants increased the likelihood of complication occurrence: undergoing surgery during 2020 (+ 67%), the increase of a unit in the AAST score (+ 26%), surgery performed > 24 h after admission (+ 58%), open surgery (+ 112%) and conversion to open surgery (+ 166%). In Italian hospitals, in March and April 2020, the number of appendectomies has drastically dropped. During the first pandemic wave, patients undergoing surgery were more frequently affected by more severe appendicitis than the previous year's timeframe and experienced a higher number of complications. Trial registration number and date: Research Registry ID 5789, May 7th, 2020

## Background

With about 60 million global infections and more than 1.5 million deaths at the end of 2020, the COVID-19 pandemic has radically changed the world [1]. Hospitals and healthcare systems had to face a significant number of infected patients needing treatment. Consequently, we significantly reduced surgical activity in the elective setting of about 30 million surgical procedures worldwide in a period of 12 weeks [2]. Nevertheless, surgeons cannot postpone emergency and oncological procedures. Therefore, they issued their recommendations [3–5] suggesting caution while performing surgery. Especially at the beginning of the pandemic, major surgical societies and colleges advised using non-operative management of appendicitis and recommended against laparoscopy [6, 7].

In the beginning, the focus was the safety of the operators [8]. Later, the potential worsening of SARS-CoV-2 pneumonia showed a high lethality rate, especially after general anaesthesia [2, 9]. Despite the growing consciousness of the phenomenon, very little attention focused on the effects of the delay in diagnosis and management of surgical diseases. A recent international web survey reported increased non-operative management of acute appendicitis during the COVID-19 outbreak [10].

The lifetime risk of acute appendicitis is 6.7% for women and 8.6% for males [11]. We estimated that about 300.000 patients in the U.S. undergo appendectomy annually, with a raw incidence of 98 cases/100,000 people [12]. Even though non-operative management is suitable for uncomplicated cases [13], laparoscopy remains the standard for treating appendicitis [14].

The study hypothesises that a significant reduction in the number of emergent appendectomies was observed during the pandemic, restricted to complex cases. The study aimed to analyse emergent surgical appendectomies during the pandemic on a national basis and compare it to the same period of the previous year.

## Methods

The CRAC study (ChiRurgia Appendiciti COVID-19, COVID-19 Appendicitis Surgery) is a national multicentre, retrospective, observational cohort study to assess the surgical outcomes of patients undergoing an appendectomy. The study compares data collected in the 2 months of March–April 2019 with those of March–April 2020. The Italian Society of Endoscopic Surgery and new technologies (SICE) endorsed the study. The protocol obtained the approval of the Ethical Committee for Clinical Trials of Treviso and Belluno on May 7, 2020 (ID: license 883/CE Marca, Italy).

One hundred fifty eight surgical units of the 448 registered in the Italian Ministry of Health registry (35.3%) adhered to the study, and 113 (71.5%) contributed to sharing data. We collected data through a Google form. The study's primary outcome was the number of appendectomies performed during each of the two months, classified according to the American Association for the Surgery of Trauma (AAST) score [15]. Secondary outcomes were the type of surgical technique (laparoscopic vs open), the number of complications classified according to the Dindo–Clavien grading system, and the mortality at 30 days [16]. Inclusion criteria were age > 18 years and occurrence of emergent appendectomy not associated with other surgical procedures. We collected gender, age, year of surgery, and Appendicitis Inflammatory Response (AIR) score [17] for each included patient. We also analysed the delay of surgery after diagnosis (< or > 24 h) and the conversion rate from laparoscopy to open. We registered hospital stay, post-operative complications classified according to Dindo–Clavien, radiological or surgical re-intervention and mortality within 30 days.

### Statistical analysis

#### Descriptive and inferential statistics

The descriptive statistics for continuous variables were reported as the median-interquartile range (IQR), while those categorical were absolute/relative frequencies. The inferential statistics, either the Mann–Whitney/Kruskal–Wallis or the Fisher’s exact test, was applied to continuous and categorical covariates, respectively. A complete set of the univariate and multivariate binary logistic regression model estimated the likelihood of a surgical complication occurrence (dependent variable in statistics, target in machine learning). We tested eight predictors (independent variables in statistics, features in machine learning) for their potential impact on complications: three continuous (age, AIR and AAST scores) and five categorical (gender, year of treatment, surgery timing, surgical technique and conversion to laparotomy) variables. AIR and AAST, despite their categorical, ordinal nature, were treated as continuous covariates due to the high number of levels (risk of over-parametrisation). We obtained all *p* values by the exact two-sided method at the conventional 5% significance level. Data were analysed as of February 2021 using R 4.0.4 packages lares version 4.9.12 and H2O version 3.32.0.4 [18].

#### Development and validation of ML models

The function h2o_automl of the R package lares was applied to access H2O for R, an open-source distributed machine learning (ML) platform [19, 20]. We trained six different supervised ML algorithms for binomial classification for target prediction (complications occurrence): GLM (Generalized Linear Model), GBM (Gradient Boosting Machine), XGBoost (Extreme Gradient Boosting machine), Distributed Random Forest (DRF), DNN (multilayer artificial Deep Neural Network) and NB (Naïve Bayes classifier) as well as two Stacked Ensemble models, one containing all the models, the second only the best from each algorithm class. We balanced the target in the training data via resampling for all models, and no missing-values replacement was needed (only four data were missing). We split the original dataset randomly for training into 80% training set and 20% test one. We used five-fold cross-validation to compare the classifiers to decrease the risk of model overfitting. We investigated the model's performance on the test set and identified the best prediction performance by the Area Under the Curve (AUC) of the Receiver Operating Characteristic (ROC) curve.

## Results

**Fig. 1:**
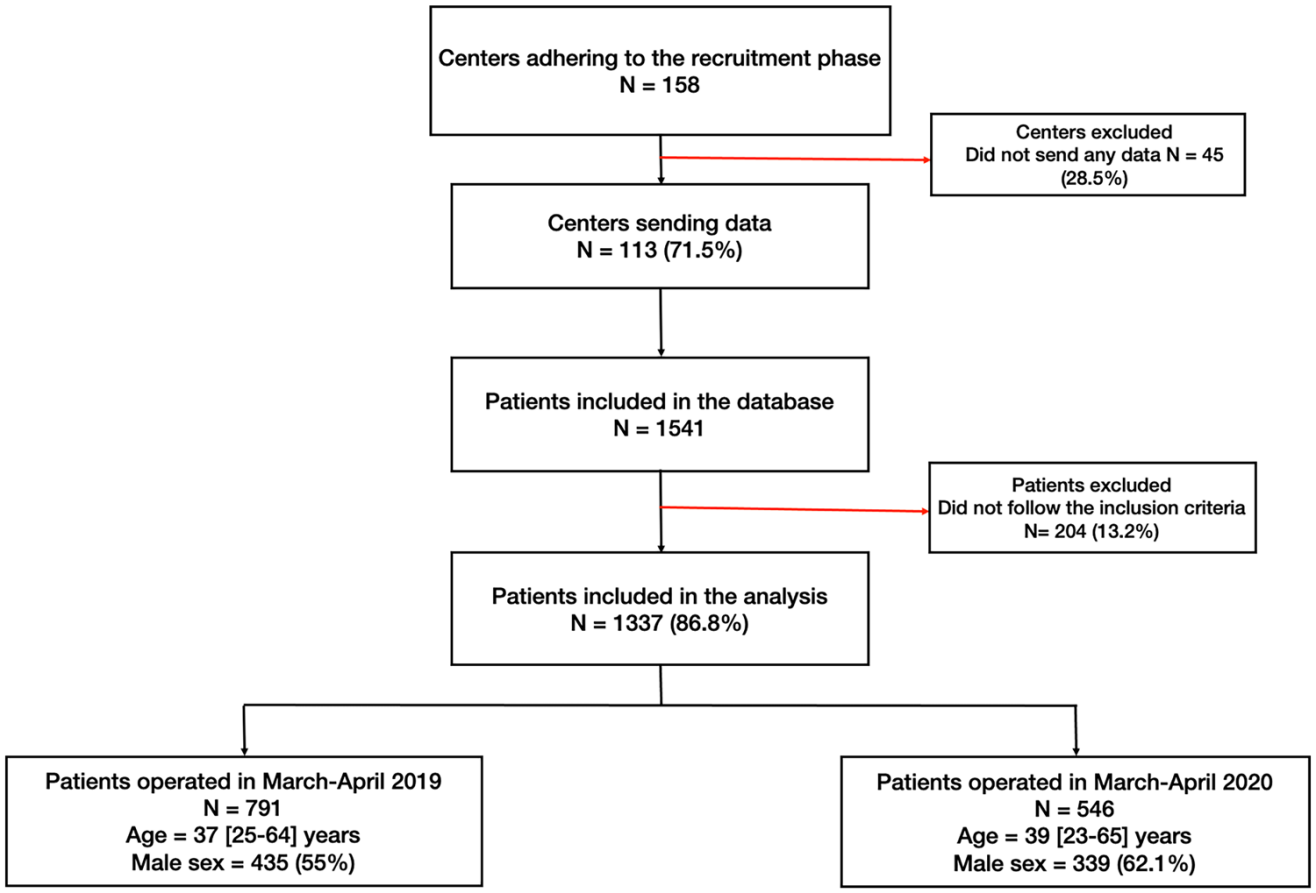
Study flow diagram

The database included 1541 patients with acute appendicitis who underwent surgery during the two study periods. According to the inclusion criteria, 1337 (86.8%) represented the study's cohort in the analysis. The study flow diagram is shown in Fig. 1.

We collected 546 patients who underwent surgery for acute appendicitis in 2020 vs 791 in 2019. Therefore, we observed a decrease in the appendectomy rate of 31%.

We summarised data about the cohort of patients stratified by year of surgery in Table 1.

**Table 1:** Patients’ characteristics: Cohort of patients stratified by year of surgery

Variable	Patients operated during 2019 (791)	Patients operated during 2020 (546)	*P*
Age: years (IQR)	37(25–64)	39 (23–65)	0.526
Gender (M vs. F): n. patients (%)	435 (55.0%)	339 (62.1%)	0.011
Complications: n. patients (%)	76 (9.6%)	87 (15.9%)	0.001
AIR score:n. patients (%)			0.036
1–4 5–8 9–12	193 (24.1%) 498 (62.9%) 100 (12.6%)	111 (20.3%) 333 (60.9%) 102 (18.6%)	
AAST score. n. patients (%)			0.008
1 2 3 4 5	379 (47.9%) 172 (21.7%) 82 (10.4%) 115 (14.5%) 43 (5.4%)	223 (40.8%) 105 (19.2%) 69 (12.6%) 112 (20.5%) 37 (6.8%)	
Surgery timing (≤ 24 vs. > 24 h): n. patients (%)	146 (18.5%)	83 (15.2%)	0.122
Surgical approach (lap vs. open): n. patients (%)	77 (9.7%)	54 (9.9%)	0.926
Conversion to open (no vs. yes): n. patients (%)	31 (3.9%)	31 (5.7%)	0.147
Dindo score: n. patients (%)			< 0.001
0 1 2 3 4 5	2 (0.3%) 664 (84.1%) 95 (12.0%) 19 (2.4%) 8 (1.0%) 2 (0.3%)	2 (0.4%) 402 (73.8%) 108 (19.8%) 29 (5.3%) 3 (0.6%) 1 (0.2%)	
Hospital stay: days (IQR)	3 (2–4)	3 (2–5)	0.722

The median age of the patients was 38 years (IQR 25–55), 37 years (IQR 25–64) for those operated in 2019 vs 39 years (IQR 23–65) for those operated in 2020 (*p* = 0.526).

The severity of appendicitis was higher among patients undergoing emergent appendectomy in 2020 vs 2019. This severity resulted in both the AIR score (*p* = 0.036) and the AAST score (*p* = 0.008).

The surgical technique used was laparoscopy in 1206 (90.2%) cases and laparotomy in 131 (9.8%). An open technique was used in 77 (9.7%) patients during 2019 vs. 54 (9.9%) during 2020 (*p* = 0.926). The conversion rate was 3.9% in 2019 vs 5.7% during 2020 (*p* = 0.147).

A thousand one hundred and eight patients underwent surgery < 24 h from admission (80.8%) while 229 (19.2%) > 24 h. In 2019, 146 (18.5%) patients had surgery delayed > 24 h from the admission vs 83 (15.2%) in 2020 (*p* = 0.122).

The median hospital stay was three days (IQR 2–5), the same as in 2019 (IQR 2–4) and in 2020 (IQR 2–5) (*p* = 0.722).

Complications were reported in 163 (12.2%) patients, while no complications occurred in 1174 patients (87.8%). Patients with higher AAST appendicitis scores had higher complication rates. We summarised the characteristics of the cohort of patients stratified according to the occurrence of post-operative complications in Table 2. The analysis of the complications according to the Dindo–Clavien grading system showed a statically significant increased rate of adverse events in 2020 (87 patients, 15.9%) compared to 2019 (76 patients, 9.6%) (*p* < 0.001).

**Table 2:** Patients’ characteristics: Cohort of patients stratified by complication occurrence

Variable	Patients without complications (1174)	Patients with complications (163)	*P*
Age: years (IQR)	37(27–63)	47 (27–62)	< 0.001
Gender (M vs. F): n. patients (%)	665 (56.6%)	109 (66.9%)	0.014
Surgery: year (2019 vs. 2020): n. patients (%)	459 (39.1%)	87 (53.4%)	0.001
AIR score: n. patients with (%)			0.003
1–4 5–8 9–12	286 (24.4%) 721 61.4%) 167 (14.2%)	18 (11.0%) 110 (67.5%) 35 (21.5%)	
AAST score: n. patients (%)			< 0.001
1 2 3 4 5	562 (47.9%) 246 (21.0%) 127 (10.8%) 178 (15.2%) 61 (5.2%)	40 (24.5%) 31 (19.0%) 24 (14.7%) 49 (30.1%) 19 (11.7%)	
Surgery timing (≤ 24 vs. > 24 h): n. patients (%)	192 (16.4%)	37 (22.7%)	0.046
Surgical approach (lap vs. open): n. patients (%)	99 (8.4%)	32 (19.6%)	< 0.001
Conversion to open (no vs. yes): n. patients (%)	43 (3.7%)	19 (11.7%)	< 0.001
Dindo score: n. patients (%)			< 0.001
1 2 3 4 5	1016 (86.8%) 141 (12.0%) 14 (1.2%) 0 (0%) 0 (0%)	54 (33.1%) 62 (38.0%) 34 (20.9%) 11 (6.7%) 2 (1.2%)	
Hospital stay: days (IQR)	3 (2–11)	7 (3–12)	< 0.001

We observed post-operative complications in 99 patients (8.4%) operated with laparoscopy, compared to 32 patients (19.6%) operated with an open technique (*p* < 0.001), and 19 patients (11.7%) who had a conversion from laparoscopy to an open procedure (*p* < 0.001).

Further treatment was necessary for 69 (5.2%) patients within 30 days from the surgical operation, of whom 15 patients (1.1%) required a radiological intervention and 54 (4%) patients required further surgery.

Three patients (0.2%) died within 30 days after surgery due to sepsis. The mortality rate was 0.25% in 2019 and 0.2% in 2020.

### Analysis of complications: univariate and multivariate models

**Fig. 2:**
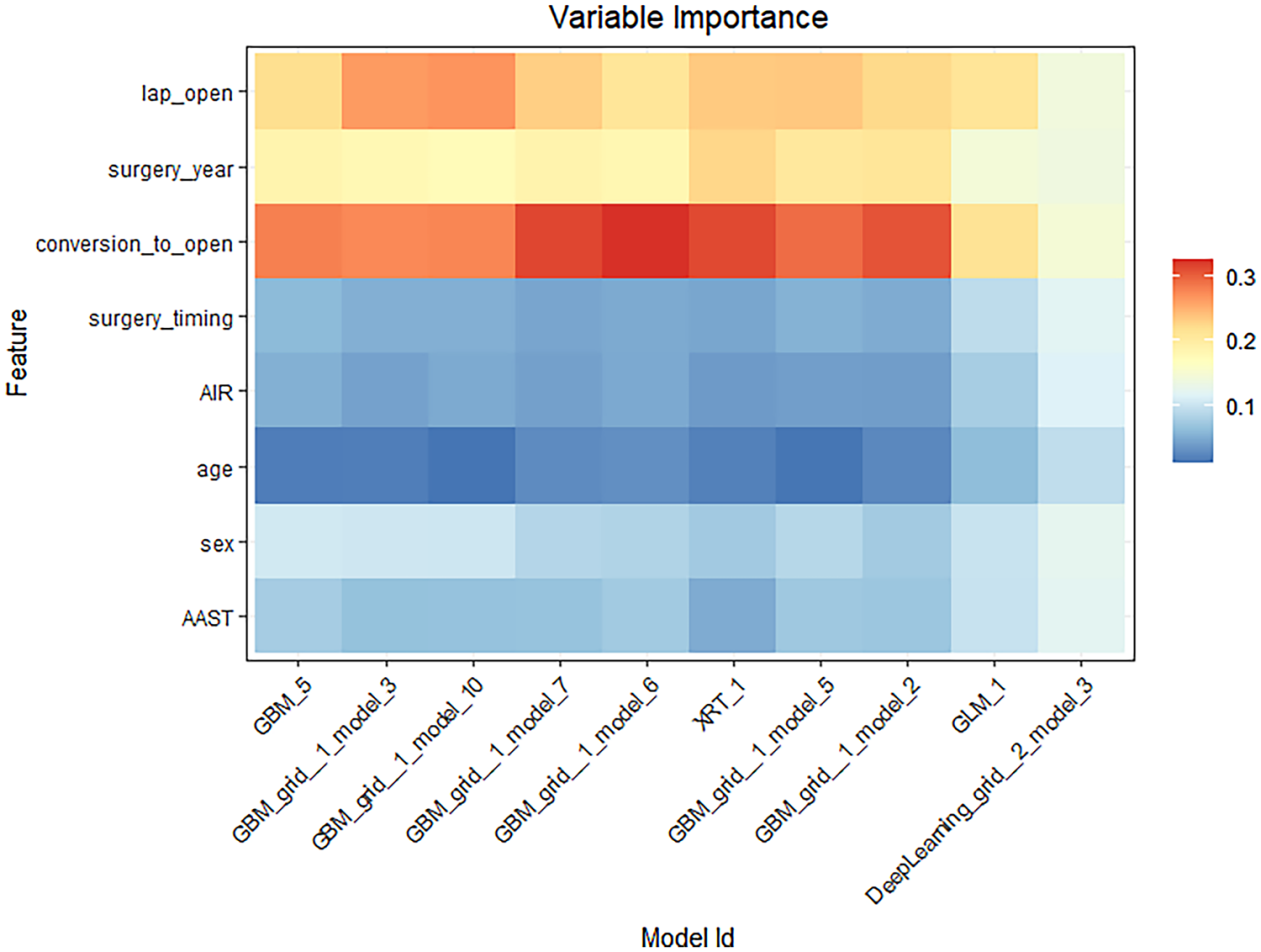
Variable importance for the top ten ML models

**Fig. 3:**
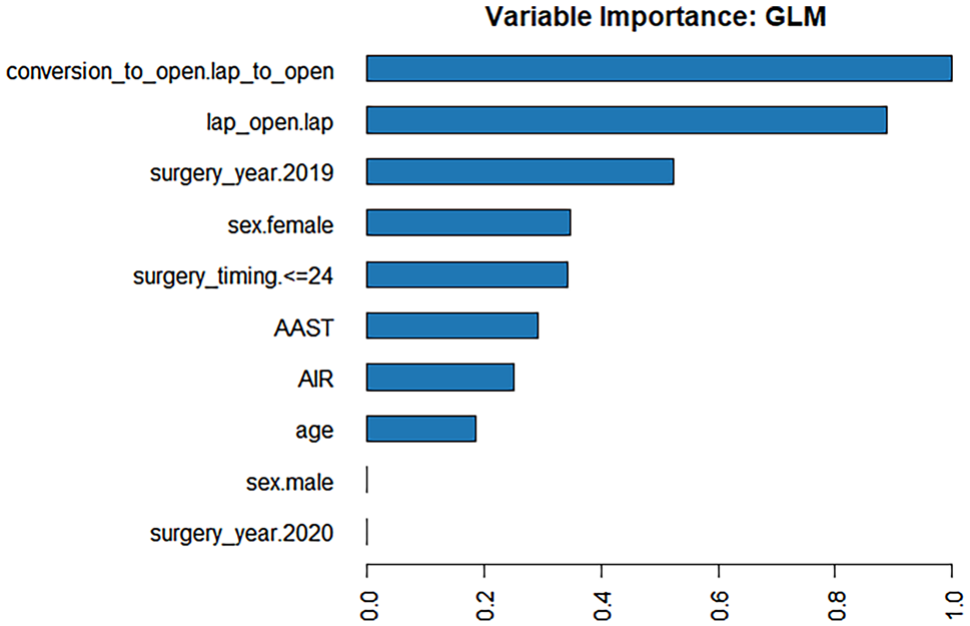
Variable importance for the best ML model (GLM)

We report the binary logistic regression model results in Table 3. In the univariate model series, all covariates played a critical role in complication occurrence, except for age and gender. The determinants that increased the likelihood of complication occurrence were undergoing surgery during 2020 (+ 64%), having a unit AIR (+ 10%) or AAST (+ 28%) increase, having waited for surgery > 24 h (+ 61%), undergoing open surgery (+ 124%) and converted to open surgery (+ 114%). The multivariate logistic model AUC was 0.720 and, when comparing it to that from ML modelling, the best AUC was obtained by the GLM model (0.724). Therefore, the ML approach fully confirmed the results obtained by the classical logistic model approach. In Figs. 2 and 3, the ranking of the features (variable importance) is plotted for the top ten models and the best one (GLM), respectively [20].

**Table 3:** Uni- and multi-variate binary logistic regression models for complication occurrence

Variable	Univariate models			Multivariate model		
	OR	95% CI	*p*	OR	95% CI	*p*
Age	1.02	1.01–1.03	< 0.001	1.01	0.99–1.02	0.139
Gender (M vs. F)	1.55	1.10–2.19	0.013	1.23	0.85–1.76	0.266
Surgery year (2020 vs. 2019)	1.78	1.28–2.47	< 0.001	1.64	1.16–2.31	0.005
AIR score	1.19	1.10–1.28	< 0.001	1.10	1.01–1.19	0.025
AAST score	1.49	1.33–1.68	< 0.001	1.28	1.11–1.47	< 0.001
Surgery timing (> 24 h vs. ≤ 24)	1.50	1.01–2.23	0.046	1.61	1.05–2.46	0.033
Surgical approach (open vs. lap)	2.71	1.75–4.20	< 0.001	2.24	1.40–3.58	0.001
Conversion to open (yes vs. no)	3.46	1.96–6.11	< 0.001	2.14	1.17–3.92	0.018

## Discussion

The CRAC study collected data from a selected cohort of Italian hospitals and showed that in March and April 2020, the number of appendectomies has drastically dropped compared to 2019. During the first wave of the COVID-19 pandemic, patients undergoing surgery were more frequently affected by more severe appendicitis forms than the previous year's timeframe, according to both the AIR and the AAST scores. The present dataset is much more comprehensive than previous international and national surveys [8, 21, 22] and multicentre retrospective studies [23, 24].

How we should interpret these data is questionable. The reduction in the overall number of appendectomies performed may have several explanations. First, individuals affected by acute appendicitis may have renounced to approach emergency rooms in hospitals for fear of SARS-CoV-2 contagion. Simultaneously, we cannot exclude that surgeon restricted the indications for surgery to more severe cases, offering milder clinical cases the opportunity of conservative medical therapy at home. Most patients with uncomplicated appendicitis require active observation and pain control [25, 26], and this is what might have happened during the first wave of the pandemic.

Since the outbreak of the COVID-19 pandemic in Europe and the U.S., much ahead, it was possible to organise a SARS-CoV-2 vaccination modelling for safe surgery [27], several recommendations issued by surgical societies and institutional bodies have supported surgery's decision-making processes, including emergency scenarios. Although the overall level of evidence of such recommendations was low, there has been a significant impact of these documents on surgeons' daily clinical practice. Globally, during the first wave of the COVID-19 pandemic, recommendations on the treatment of acute appendicitis suggested the use of appropriate non-operative treatments whenever possible to avoid overloading hospitals, already heavily burdened by SARS-CoV-2 patients. Our findings align with the ACIE Appy international survey on the global attitudes in managing acute appendicitis during the pandemic. These showed a statically significant decrease in the number of acute appendicitis patients referred to the hospitals, with only 10% of surgical units reporting > 20 referrals per month [10]. In the present study, while the absolute numbers of cases AIR score > 8 and AAST > 3 remained constant between the two years, a significant reduction in the milder cases was observed.

The second recommendation focused on the technique to adopt. Initial guidelines recommended open appendectomy in case of intra-abdominal sepsis or non-resolving disease following antibiotic [28–30]. Former detection of activated viruses (corynebacterium, papillomavirus, HBV, HIV) in the surgical smoke suggested that SARS-CoV-2 might behave similarly [31]. This observation inspired suggestions to avoid the use of laparoscopy. We must consider such recommendations in the existing scenario. Here, European surgeons faced the viral spread during the first wave of the pandemic, together with the lack of ultrafiltration systems, personal protective equipment, surgical workforce and routine testing of patients. However, one year after identifying the SARS-CoV-2 infection in China, an active virus has not been isolated so far from the laparoscopic plumes within the peritoneal cavity of infected subjects.

Consequently, the potential of viral spreading during laparoscopy is not known. Based on clinical judgment, surgeons should prefer a laparoscopic appendectomy when resources are available. The safe performance of laparoscopic appendectomy allows for short hospitalisation. Data from the CRAC study showed that, in Italy, the rate of laparoscopic appendectomy performed in March–April 2020 was comparable to that in the same two months in 2019. This finding contrasts with the trend in favour of open appendectomy reported in other countries [32]. Laparoscopy should always be preferred as it provided, also in this study, a lower rate of complications compared to both open surgery and conversion to open surgery. The high rate of laparoscopy is a consequence of the effort to equip operating rooms with systems for the safe evacuation of laparoscopic plumes. This effort included first homemade systems [33], then the certified ones, following the adoption of the EAES guidelines [3, 34]. Even the rate of interventions converted from laparoscopy to open surgery did not increase significantly. This can be justified by the higher complexity of the cases, as reported by AIR and AAST scores.

As the severity of appendicitis among the operated patients significantly increased during the first wave of the pandemic, this correlated with increased complication rate and severity, but not mortality. Different from perception, the circumstances did not delay admission to the emergency room. Despite the apparent difficulties in hospitals' emergency area organisation, this did not affect the surgical activity's efficiency. Nevertheless, complications observed were significantly more in 2020, as well as their severity. We observed an increase in acute appendicitis complicated with phlegmon, abscess, or diffuse peritonitis. These correspond to grades 3–5 of the American Association for the Surgery of Trauma (ASST) classification. Here, patients presenting with perforated appendicitis increased from 30.3% in 2019 to 39.9% in 2020. The CRAC study showed that those patients undergoing surgery > 24 h after observation experienced more post-operative complications. This finding aligns with the United Kingdom National Surgical Research Collaborative study on a cohort of more than 2500 patients with acute appendicitis. Here, delaying appendectomy for over 48 h was related to a statically significant increased risk of surgical-site infection and 30-day adverse events [35]. Similarly, Alore showed that patients undergoing appendectomy three days after the admission had increased 30-day morbidity and mortality [36]. In our study, the rate of complicated appendicitis reported during the pandemic period of 2020 was higher than that usually found during 2019, and generally, in contemporary literature. Therefore, we argue that it may be reasonable to prioritise patients reporting symptoms lasting > 24 h for operative management.

We must interpret our results within the context of some limitations. First, due to the urgent need for evidence on appendicitis management during the first wave of the COVID-19 pandemic, we performed only a short-term follow-up. The study design did not allow us to assess post-operative visits after 30 days from the surgical intervention. As a consequence, we might miss long-term complications, such as adhesions and incisional hernias. Second, due to the observational design, the quality of data collected depended on the quality of medical records and the researcher's interpretation of charted notes. Third, there is a considerable variation in the organisation of the emergency surgical departments across the country [37, 38]. The most relevant source of bias is probably the heterogeneity of the various centres involved in diagnostic pathways. Fourth, most likely, the reduction in appendectomies performed corresponds to an increase in non-operative management. Unfortunately, we do not have data about the non-operative management of acute appendicitis cases. Ultimately, the study has a non-randomised nature associated with any extensive database. Conclusions from non-randomised studies can be misleading because there is always a chance for selection bias, leading to underestimating or overestimating the real intervention effect. On the other hand, our study's strength lies in the fact that we demonstrated that fewer patients sought medical attention during the lock-down due to the COVID-19 pandemic acute appendicitis in the analysis of individual patients' data in Italy. Here, complicated appendicitis rates increased, leading to a higher incidence of complications than in the past.

## Conclusion

During the first wave of the COVID-19 pandemic, the number of appendectomies has drastically dropped in Italy. While patients with severe appendicitis remained constant, we observed a substantial reduction in milder cases undergoing surgery. Consequently, we observed a higher complication rate, despite mitigated by an unchanged high rate of use of laparoscopy.

## Data Availability

The datasets analyzed during the current study are available from the corresponding author on reasonable request.

## Abbreviations

COVID-19: Coronavirus disease 2019
AAST: American Association for the Surgery of Trauma
CRAC: ChiRurgia Appendiciti COVID-19, COVID-19 Appendicitis Surgery
SICE: Italian Society of Endoscopic Surgery and new technologies
AIR Score: Appendicitis Inflammatory Response Score
IQR: Interquartile Range
ML: Machine Learning
GLM: Generalised Linear Model
GBM: Gradient Boosting Machine
XGBoost: Extreme Gradient Boosting machine
DRF: Distributed Random Forest
DNN: Multilayer artificial Deep Neural Network
NB: Naïve Bayes classifier
AUC: Area Under the Curve
ROC: Receiver Operating Characteristic
